# DNA binding kinetics of two response regulators, PlnC and PlnD, from the bacteriocin regulon of *Lactobacillus plantarum *C11

**DOI:** 10.1186/1471-2091-10-17

**Published:** 2009-06-11

**Authors:** Daniel Straume, Rune F Johansen, Magnar Bjørås, Ingolf F Nes, Dzung B Diep

**Affiliations:** 1 Laboratory for Microbial Gene Technology, Department of Chemistry, Biotechnology and Food Science, Norwegian University of Life Sciences, PO Box 5003, N-1432 Ås, Norway; 2 Centre for Molecular Biology and Neuroscience, Institute of Medical Microbiology, University of Oslo, Rikshospitalet, N-0027 Oslo, Norway

## Abstract

**Background:**

Bacteriocin production in the lactic acid bacterium *Lactobacillus plantarum *C11 is regulated through a quorum sensing based pathway involving two highly homologous response regulators (59% identity and 76% similarity), PlnC as a transcriptional activator and PlnD as a repressor. Previous *in vitro *studies have shown that both regulators bind, as homodimers, to the same DNA regulatory repeats to exert their regulatory functions. As the genes for these two proteins are located on the same auto-regulatory operon, hence being co-expressed upon gene activation, it is plausible that their opposite functions must somehow be differentially regulated, either in terms of timing and/or binding kinetics, so that their activities do not impair each other in an uncontrolled manner. To understand the nature behind this potential differentiation, we have studied the binding kinetics of the two regulators on five target promoters (P_*plnA*_, P_*plnM*_, P_*plnJ*_, P_*plnE *_and P_*plnG*_) from the bacteriocin regulon of *L. plantarum *C11.

**Results:**

By using surface plasmon resonance spectroscopy we obtained parameters such as association rates, dissociation rates and dissociation constants, showing that the two regulators indeed differ greatly from each other in terms of cooperative binding and binding strength to the different promoters. For instance, cooperativity is very strong for PlnC binding to the promoter of the regulatory operon (P_*plnA*_), but not to the promoter of the transport operon (P_*plnG*_), while the opposite is seen for PlnD binding to these two promoters. The estimated affinity constants indicate that PlnC can bind to P_*plnA *_to activate transcription of the key regulatory operon *plnABCD *without much interference from PlnD, and that the repressive function of PlnD might act through a different mechanism than repression of the regulatory operon.

**Conclusion:**

We have characterised the DNA binding kinetics of the two regulators PlnC and PlnD from the bacteriocin locus in *L. plantarum *C11. Our data show that PlnC and PlnD, despite their strong homology to each other, differ greatly from each other in terms of binding affinity and cooperativity to the different promoters of the *pln *regulon.

## Background

Many lactic acid bacteria produce a group of ribosomally synthesised antibacterial peptides, most frequently referred to as bacteriocins, which kill other bacteria by disrupting the integrity of the membrane of target cells leading to leakage of cellular solutes across the membrane and eventually cell death [1-3]. Most bacteriocins have quite narrow inhibitory spectra, normally composed of species closely related to the bacteriocin producers. This feature is believed to give the producers an advantage within certain ecological niches in competition for common resources [4]. In many cases, bacteriocin production is regulated through a quorum sensing pathway mediated by a secreted inducer peptide (IP), a transmembrane sensor histidine protein kinase (HPK) and a cytoplasmic response regulator (RR) [4,5]. The IP is believed to be initially produced at a low constitutive level during early exponential growth phase, but can reach a critical threshold concentration, either by a cumulative process via constitutive production or by elevated production triggered by some environmental cues, e.g., co-cultivation with other bacterial strains [6-8]. When the critical threshold level of IP is achieved, this signal is processed by the IP-binding protein HPK and subsequently transferred to its cognate RR via a series of phosphorylation reactions that eventually results in a phosphorylated RR. The latter binds as dimers to regulated promoters and triggers expression of all genes involved in bacteriocin biosynthesis [5,9-11].

**Figure 1:**
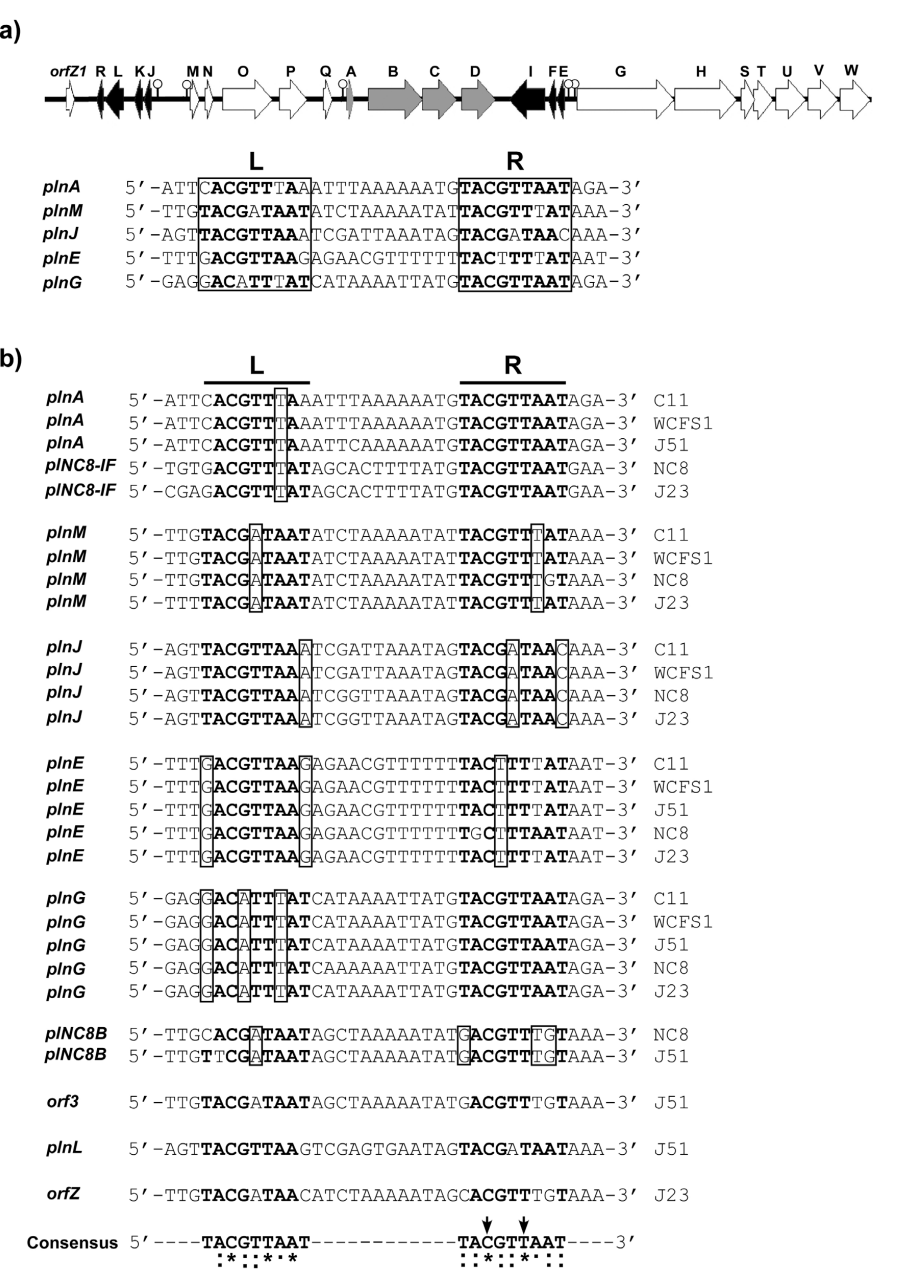
**The *pln *locus in *L. plantarum *C11 and sequence alignment of the two tandem repeats located in the *pln *promoters of five different strains of *L. plantarum *(C11, WCFS1, NC8, J23 and J51)**. (a) Genetic organisation of the *pln *locus in *L. plantarum *C11 and sequences of the conserved tandem repeats (left (L) and right (R) repeat) involved in regulation of the *pln *operons. Lollipops indicate the position of the regulatory repeats. (b) The regulatory repeats from all five *pln *loci organised based on which operons they control. Conserved nucleotides are shown in bold letters and non-consensus nucleotides that are conserved within the functional groups are boxed. Nucleotides in the two tandem repeats that are 100% conserved are marked with an asterisk, and highly conserved and moderately conserved nucleotides are marked with two dots and one dot, respectively. The two arrows indicate nucleotides that have been shown to be crucial for binding of both PlnC and PlnD [22].

Bacteriocin production in *Lactobacillus plantarum *C11 is regulated by such a quorum sensing network. Its bacteriocin locus, called the plantaricin locus (*pln*), is organised in five operons (see Figure 1a): *plnEFI *and *plnJKLR *code for two two-peptide bacteriocins and their cognate immunity proteins, *plnGHSTUVW *contains two genes (*plnGH*) that code for a complete ABC-transporter system dedicated to export peptides containing a so-called double-glycine leader, while *plnTUVW *codes for type II CAAX proteases [12] with unknown function in bacteriocin biosynthesis, *plnABCD *for a regulatory network and *plnMNOP *for proteins with unknown functions [9]. In the regulatory operon, *plnA *encodes a secreted IP, *plnB *an HPK, while the last two genes, *plnCD*, encode two highly homologous RRs belonging to the LytTR family [13,14] whose members contain a DNA-binding domain lacking the typical helix-turn-helix motif. PlnC and PlnD are both 247 amino acids in length, and share remarkably high homology to each other (59% identity and 76% similarity in the amino acid sequence). Previous studies have demonstrated that over-expression of *plnC *in the endogenous host activates the genes involved in bacteriocin production, while over-expression of *plnD *represses the same set of genes [15,16], suggesting that these two proteins probably possess different roles in gene expression, i.e., PlnC being a transcriptional activator while PlnD acts as a repressor. The involvement of two homologous RRs in a quorum sensing network is a rare phenomenon in bacteriocin production as well as in other processes such as competence development, biofilm formation and production of toxins [17-19]. Interestingly, two other strains of *L. plantarum*, NC8 and J23, each contain a bacteriocin locus very similar to that of C11, but surprisingly, their individual regulatory operons encode only one RR, namely a PlnD homologue (97 and 98% homologous to PlnD in C11, respectively), which most probably acts as an activator of bacteriocin production in these strains [20,21].

*In vitro *studies have shown that both PlnC and PlnD bind to the same set of regulated promoters, each of which is composed of two conserved 9-basepair (bp) tandem repeats and a 12 bp intervening spacer region, located directly upstream of the -35 and -10 boxes (Figure 1a). The 12-bp spacer corresponds to approximately one DNA helical turn, thus facing the pairwise repeats towards the same side of the DNA molecule, a feature probably necessary for cooperative dimeric binding with one regulator bound to each DNA repeat. Mutation studies have indeed confirmed that both the tandem repeats and the 12-bp length of the spacer are crucial for dimeric binding of the *pln *RRs [11,22].

With regard to their co-expression (both located within the same operon) and their opposite functions, it is not known how the activator PlnC and the repressor PlnD coordinate their actions when they regulate gene expression from the same set of promoters. Our hypothesis is that these two regulators might differentiate from each other in their binding kinetics and/or mode of dimeric cooperativity to the different promoters. We have therefore undertaken a DNA binding kinetics study on PlnC and PlnD using surface plasmon resonance (SPR) spectroscopy, and determined their dissociation constants to all five regulated promoters. Our study reveals that these two regulators indeed show marked differences in their binding kinetics. How these results relate to their biological functions will be discussed.

## Results

### DNA sequence analysis of the promoter-associated regulatory motifs from the different *pln *loci

In addition to the strain C11, four other strains of *L. plantarum *(NC8, WCFS1, J23 and J51) each have been reported to harbour a similar *pln *bacteriocin locus in their genomes. Among these strains, the *pln *loci of WCFS1 and J51 (hereafter called WCFS1-*pln *and J51-*pln *and similar designations used for the other *pln *loci) are more similar to C11-*pln *with regard to the regulatory operon, all three involving two RRs (PlnC and PlnD), while the other two loci (NC8-*pln *and J23-*pln*) involve only one RR, a PlnD-like protein. Furthermore, all five *pln *loci show different degrees of similarity in terms of sequence similarity and gene content in the other operons, with highest variation in the *plnMNOP *and *plnJKLR *operons (see additional file 1).

*In silico *analyses have suggested that the regulated promoters from the other *pln *loci are regulated in the same manner as in C11-*pln *since the regulated promoters from all these *pln *loci are built up in a similar fashion, i.e., each containing a pair of tandem repeats located just upstream of the -35 and -10 boxes, and the consensus sequence (5'-TACGTTAAT-3') of the repeats is the same as that found in the C11-*pln *locus [7,21,23]. For C11-*pln*, the right repeats appear to be more conserved than the left repeats; the five right repeats together contain only five nucleotides different from the consensus sequence while the left repeats together contain 10 such variant nucleotides. Another interesting feature worth mentioning is that while most repeats contain at least one variant nucleotide, the right repeats of the promoters P_*plnA *_and P_*plnG *_are invariant (the former associated to the regulatory operon while the latter to the transport operon). On the other hand, the left repeats of these two promoters are most degenerated from the consensus sequence, i.e., both containing three variant nucleotides (Figure 1b).

Interestingly, the distribution of sequence variations within the regulatory repeats seems to be relatively conserved within promoters belonging to the same functional groups. This can be seen in Figure 1b where the tandem repeats from the different loci are organised based on which operons they control. For instance, the right repeats in the promoters of the regulatory operons (P_*plnA *_and P_*plNC*8-*IF*_) and the transport operons (P_*plnG*_) are invariant in all loci. Similarly, most variant nucleotides in the other repeats are conserved within the individual functional groups. Also the sequence of the spacers is highly conserved within the different functional groups. Together, the high degree of conservation of the variant nucleotides suggests that these differences probably have important roles in the regulation of bacteriocin biosynthesis.

Furthermore, the alignment of the promoters from all five *pln *loci revealed that the left and right repeats contain three (C_3_, T_6 _and A_8_) and two (C_3 _and T_6_) nucleotides, respectively, that are 100% conserved, indicating that these are crucial for binding of the RRs. This is consistent with previous gel-shift studies which showed that substitutions of C_3 _and T_6 _in the right repeat almost abolished binding of PlnC and PlnD [22]. It also seems that the right repeat in general is more conserved than the left repeat, mainly because the first and last nucleotides in the left repeats show relatively high variation frequency.

### Binding of f-PlnC and f-PlnD to the regulated *pln *promoters in *L. plantarum *C11

**Figure 2:**
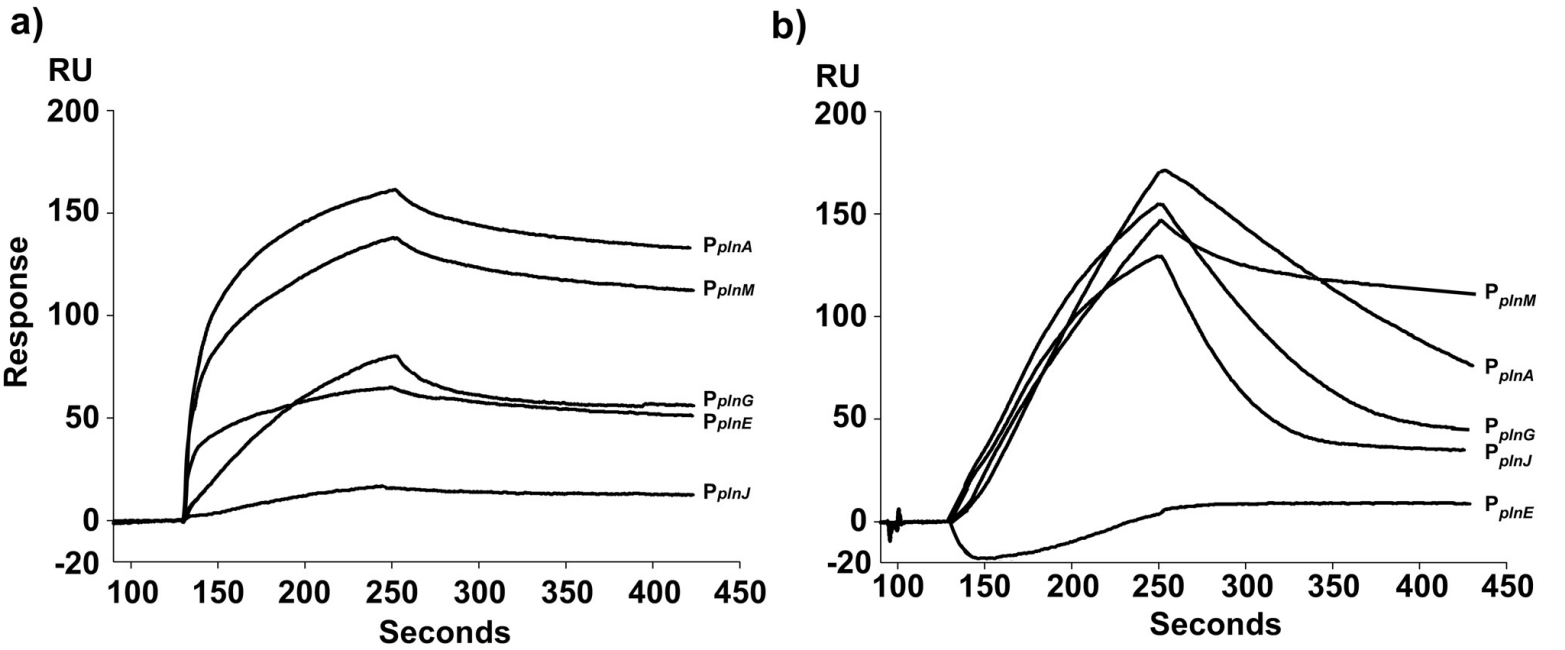
**SPR analysis of the interaction between f-PlnC or f-PlnD and the regulated promoters in the C11-*pln***. f-PlnC (a) and f-PlnD (b) were allowed to associate to the promoters P_*plnA*_, P_*plnM*_, P_*plnG*_, P_*plnE *_and P_*plnJ *_for 120 seconds at 25°C followed by injection of running buffer for 180 seconds for dissociation. All curves were corrected for bulk refractive index change and non-specific binding to the control DNA. The regulators were injected to the flow cells at a concentration of 1000 nM.

To study the binding kinetics of PlnC and PlnD, we took advantage of their N-terminally flag-tagged versions f-PlnC and f-PlnD. This tag facilitates purification by immunoprecipitation, and these fusion proteins have previously been proven to retain the same functionality as their wild-type counterparts in gene regulation [15]. To determine whether f-PlnC and f-PlnD bind the promoters differently, we first examined their association and dissociation profiles to the five regulated promoters from the C11-*pln *locus (P_*plnA*_, P_*plnM*_, P_*plnE*_, P_*plnJ *_and P_*plnG*_, see Figure 1a and Table 1) using SPR spectroscopy. Figure 2 shows that the sensorgrams conferring f-PlnC binding to P_*plnA*_, P_*plnM *_and P_*plnE *_climbed more sharply in the association phase than that of f-PlnD, indicating that f-PlnC has more rapid association kinetics than f-PlnD for these promoters. f-PlnC also appeared to reach binding equilibrium more quickly than f-PlnD for these three promoters as judged by the decreased slope of the f-PlnC sensorgrams at the end of the association phase. In contrast, f-PlnC seemed to associate slower than f-PlnD to P_*plnG *_and P_*plnJ*_. With regard to the dissociation phase, the sensorgrams indicate that f-PlnC dissociates slower from all promoters, except for P_*plnM *_from which dissociation was more or less similar to that of f-PlnD. These results clearly demonstrate that f-PlnC and f-PlnD have different binding properties to the five *pln *promoters in terms of both association and dissociation kinetics.

**Table 1:** DNA sequences of regulatory elements in the *pln *promoters used for SPR spectroscopy studies.

Promoter	Sequence ^a^L ^a^R
P_*plnA*_	5'-CATGGTGATTC**ACGTT**T**A**AATTTAAAAAATG**TACGTTAAT**AGAAATAATT-3'
P_*plnM*_	5'-TGAATTATTG**TACG**A**TAAT**ATCTAAAAATAT**TACGTT**T**AT**AAAAATATCG-3'
P_*plnJ*_	5'-ACTTTCAAGT**TACGTTAAA**TCGATTAAATAG**TACG**A**TAA**CAAATTTAAAA-3'
P_*plnE*_	5'-ATTGGTATTTG**ACGTTAA**GAGAACGTTTTTT**TAC**T**TT**T**AT**AATTTTTTCA-3'
P_*plnG*_	5'-GCCTGATGAGG**AC**A**TT**T**AT**CATAAAATTATG**TACGTTAAT**AGATAGTTGG-3'
P_*plnAΔL*_	5'-CATGGTGATTT*TAAAATT*TATTTAAAAAATG**TACGTTAAT**AGAAATAATT-3'
^*b*^Control DNA	5'-CATGGTG*TCG***CA***A***GT***G***T***T***A***CGG*TAAAAA*GAC***TA***A***GT***G***A***T***T***CCC*AATAATT-3'

^*a *^The left and right repeats (L and R) are underlined and conserved nucleotides within the repeats are shown in bold letters. Shown here are the forward sequences.

^*b *^Nucleotide substitutions are shown in italic.

Moreover, the sensorgrams also show that the five promoters have different degrees of binding capacity for the two RRs. Both regulators produced highest sensor signals when binding to their own promoter (P_*plnA*_), (160–170 response units, RU), whereas the signals were very poor for f-PlnC to P_*plnJ *_(less than 20 RU), and hardly detectable for f-PlnD to P_*plnE*_.

### DNA binding kinetics of f-PlnC and f-PlnD

**Figure 3:**
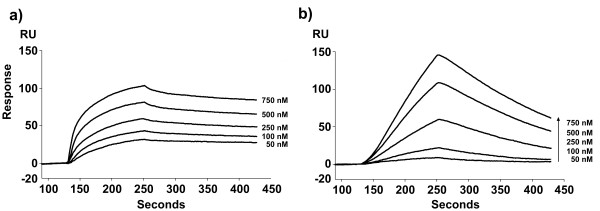
**Binding of f-PlnC and f-PlnD to P_*plnA*_**. The sensor response curves show the interaction between f-PlnC (a) or f-PlnD (b) and P_*plnA *_at protein concentrations of 50, 100, 250, 500 and 750 nM. Association was allowed to proceed for 120 seconds at 25°C with subsequent dissociation by injecting running buffer for 180 seconds. All curves were corrected for bulk refractive index change and non-specific binding to the control DNA before estimating the binding kinetics.

Next we used SPR spectroscopy to determine the binding kinetics of the two RRs towards the different *pln *promoters. Five concentrations of f-PlnC or f-PlnD (50, 100, 250, 500 and 750 nM) were introduced to the sensor chip pre-immobilised with the individual promoter sequences. The overall sensor response increased in a dose-dependent manner as demonstrated for binding to P_*plnA *_in Figure 3. Similar sensorgrams were obtained for the other promoters (data not shown). The fitting model called heterogeneous ligand – parallel reactions (two different binding sites on the ligand that each bind one analyte molecule) provided the best fit for the DNA-sequences (ligand) containing two conserved repeats (binding sites), while the Langmuir 1:1 model (independent binding between ligand and one analyte molecule) generated the best fit for P_*plnA*Δ*L*_, which contains only one binding site. Residual values ranged within ± 10 RU and the chi-square values (χ^2^) from 10 to below 1 (data not shown). The estimated dissociation constants, represented by K_D_, are quantitative parameters describing the strength of the interaction between the regulator molecule and the target site. A low K_D _indicates a strong interaction and vice versa. Since each *pln *promoter contains two binding sites (each site binding one regulator molecule), two dissociation constants, K_D1 _and K_D2_, were obtained representing the affinity for site 1 and site 2, respectively. However, it should be underlined that the fitting model used does not take into account whether site 1 or site 2 represents the left or the right repeat on the DNA. The affinity constants that were calculated from the SPR spectroscopy measurements clearly show that f-PlnC and f-PlnD display different affinities for the various promoters (Table 2). It is also evident that both RRs in most cases bound considerably stronger to site 2 compared to site 1, probably a consequence of cooperativity (see below).

**Table 2:** DNA binding kinetics and affinities of f-PlnC and f-PlnD.

Analyte	Ligand	Ka_1_ (M^-1^s^-1^)	Kd_1_ (s^-1^)	Ka_2_ (M^-1^s^-1^)	Kd_2_ (s^-1^)	^*a*^K_D1_ (M)	K_D2_ (M)	K_D1_/K_D2_
f-PlnC	P_*plnA*_	6.8 ± 6.1 × 10^2^	1.0 ± 0.1 × 10^-3^	1.5 × 10^5^	1.0 × 10^-3^	1.5 ± 5.3 × 10^-6^	6.7 ± 0.1 × 10^-9^	2.2 × 10^2^
	P_*plnM*_	3.9 ± 0.9 × 10^3^	1.5 ± 0.1 × 10^-3^	2.3 ± 0.1 × 10^5^	7.5 ± 0.5 × 10^-4^	3.8 ± 0.6 × 10^-7^	3.2 ± 0.3 × 10^-9^	1.2 × 10^2^
	P_*plnJ*_	NA	NA	NA	NA	NA	NA	
	P_*plnE*_	1.2 ± 0.9 × 10^4^	2.5 ± 0.1 × 10^-3^	2.4 ± 0.1 × 10^5^	2.6 ± 0.5 × 10^-4^	2.1 ± 0.2 × 10^-7^	1.1 ± 0.3 × 10^-9^	1.9 × 10^2^
	^*b*^P_*plnG*_	8.7 ± 7.1 × 10^3^	1.5 ± 0.4 × 10^-3^	1.3 ± 0.6 × 10^4^	1.3 ± 0.1 × 10^-3^	1.7 ± 3.9 × 10^-7^	1.0 ± 0.5 × 10^-7^	1.7
	P_*plnAΔL*_	3.2 ± 1.2 × 10^4^	9.6 ± 0.1 × 10^-3^			3.0 ± 1.3 × 10^-7^		
								
f-PlnD	P_*plnA*_	3.4 × 10^3^	5.0 × 10^-3^	3.4 ± 0.1 × 10^3^	5.1 ± × 10^-3^	1.5 × 10^-6^	1.5 ± 0.1 × 10^-6^	1.0
	P_*plnM*_	3.9 × 10^3^	2.2 × 10^-3^	1.4 × 10^4^	1.1 ± 0.3 × 10^-6^	5.6 × 10^-7^	7.8 ± 0.3 × 10^-11^	7.2 × 10^3^
	P_*plnJ*_	9.5 ± 0.3 × 10^2^	1.8 ± 0.1 × 10^-2^	2.3 ± 0.1 × 10^2^	2.2 ± 0.3 × 10^-5^	1.2 ± 0.4 × 10^-5^	9.5 ± 1.4 × 10^-8^	1.3 × 10^2^
	P_*plnE*_	NA	NA	NA	NA	NA	NA	
	P_*plnG*_	1.1 ± 0.1 × 10^3^	1.5 ± 0.1 × 10^-2^	2.5 ± 0.1 × 10^2^	9.3 ± 8.7 × 10^-6^	1.4 ± 1.1 × 10^-5^	3.7 ± 2.3 × 10^-8^	3.8 × 10^2^
	P_*plnAΔL*_	NA	NA	NA	NA	NA	NA	

^*a *^K_D1 _= *kd*_1_/*ka*_1_, K_D2 _= *kd*_2_/*ka*_2_

^*b *^The *ka *was fitted locally

NA: not available

The K_D1 _values of f-PlnC for the different promoters vary in the range of 10^-6 ^to 10^-7 ^M except for P_*plnJ *_which K_D _values could not be estimated due to very poor binding. The affinity of f-PlnC to the second binding site appeared, for most promoters, approximately 100–200 times stronger compared to the affinity for site 1 (i.e., K_D2 _values being about 100–200 times lower than the K_D1 _values), whereas for P_*plnG*_, f-PlnC seemed to bind site 2 with an affinity of about the same strength as site 1, thus both K_D _values being in the range of 10^-7 ^M. Compared to f-PlnC, f-PlnD seems to bind more differently to the *pln *promoters. Based on the K_D1 _values, f-PlnD binding to site 1 appeared to be strongest for P_*plnM *_(10^-7 ^M) and P_*plnA *_(10^-6 ^M), but somewhat weaker for P_*plnJ *_and P_*plnG*_, the last two with K_D1 _values in about the same range, 10^-5 ^M. The K_D2 _values indicate that f-PlnD associated to the second binding site of P_*plnA *_with the same affinity as for site 1 (K_D2 _at 10^-6 ^M), while it bound much stronger to site 2 of the remaining promoters, with P_*plnM *_being the strongest (K_D2 _at 10^-11 ^M). In addition, while f-PlnC associated poorly to the bacteriocin promoter P_*plnJ*_, f-PlnD showed very poor binding to the other bacteriocin promoter, P_*plnE *_(affinity constant not estimated).

**Figure 4:**
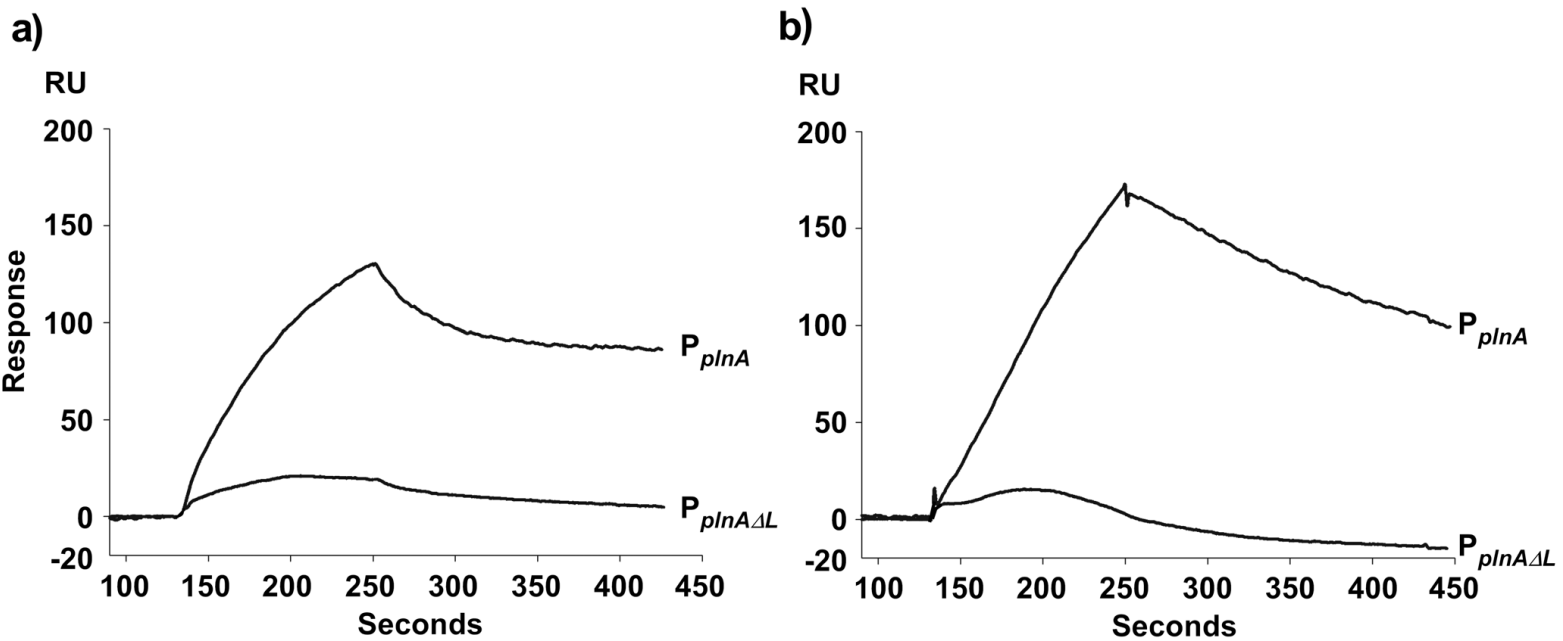
**Sensorgrams showing the difference between monomeric and dimeric binding of f-PlnC and f-PlnD**. Amounts of 1000 nM f-PlnC (a) or f-PlnD (b) were allowed to bind DNA containing only one consensus right repeat (P_*plnA*Δ*L*_), as well as to DNA containing two tandem repeats; one imperfect left repeat and one consensus right repeat (P_*plnA*_).

Tandem regulatory repeats are relatively common in regulated promoters as they together facilitate strong promoter binding via a mechanism called cooperative binding. To examine how important the tandem repeats are for cooperativ binding of the RRs in the *pln *regulon, we deleted one of the repeats from the promoter. For this experiment, we chose P_*plnA *_as it contains a right repeat of the consensus sequence (5'-TACGTTAAT-3'), which has been shown to function as the optimal site for binding of both RRs [22], and a left repeat which is highly degenerated from the consensus sequence. The left repeat was deleted resulting in P_*plnA*Δ*L*_, and binding assays were performed and compared with the wild-type promoter. As illustrated in Figure 4, the binding of f-PlnC and f-PlnD was drastically reduced (84–90%) for P_*plnA*Δ*L*_. In addition, the affinity constant of f-PlnC binding to the perfect site on P_*plnA*Δ*L *_(monomeric binding) was estimated to be in the range of 10^-7 ^M, which is similar to the affinity constants displayed for site 1 of the wild type promoter, but significantly weaker than the affinity displayed for site 2 (in the range of 10^-9 ^M). Based on these results we conclude that both repeats in the *pln *promoters are important for strong binding of the regulators and that the regulators bind on these sites as dimers in a cooperative manner.

## Discussion

The two RRs PlnC and PlnD are key components in a quorum sensing based regulatory system responsible for bacteriocin production in *L. plantarum *C11. They bind to the same set of promoters, but appear to pose activities opposite to each other, with PlnC functioning as a gene activator and PlnD as a repressor [11,15,16]. Since previous studies have shown that these two regulators are co-expressed and bind to the same promoter sequences [9,11], it is reasonable to believe that they must compete for the same binding sites in order to execute their functions. The present study was therefore conducted to reveal differences that might contribute to differentiate their regulatory activities. Indeed, although the two regulators have a number of binding properties in common, important differences between them also exist.

Based on the estimated dissociation constants, it became clear that both RRs bound significantly stronger to site 2 compared to site 1 for most of the promoters. It has previously been established that upon promoter binding, the individual regulator binds as dimers to the tandem repeats on each promoter, with one regulator molecule on each repeat. It is logical to think that the first regulator molecule, which can choose between two independent binding sites, will associate to the best suited repeat before the second regulator molecule associates to the second less suited repeat. We have here demonstrated that the monomeric binding of f-PlnC to the best suited binding site occurs with approximately 50–250 times lower affinity than binding to site 2 of P_*plnA*_, P_*plnM *_and P_*plnE*_, strongly suggesting that the higher affinity for site 2 upon dimeric binding is an effect of cooperativity. For P_*plnA*_, previous gel-shift assays have shown that PlnC and PlnD possess nearly no monomeric binding to the left imperfect repeat, while both RRs indeed bound as monomers to its consensus right repeat [22]. The previous gel-shift data combined with our new findings suggest that the RRs first bind to the consensus right repeat of P_*plnA*_, followed by a cooperative recruitment of a second regulator molecule to its imperfect left repeat. This model is strengthened by the fact that the right repeat seems to be more conserved than the left repeat among the different *pln *promoters (see Figure 1b).

The *plnABCD *operon is the key player in the regulatory circuit since the encoded proteins from this operon control expression of the entire *pln *locus; in particular the interaction between the regulatory elements of P_*plnA *_and PlnC is crucial for this process. The dissociation constants estimated for f-PlnC binding to the key promoter P_*plnA *_show that the K_D1 _value is in the range of 10^-7 ^M. More importantly, the binding of f-PlnC to the second site, measured by the K_D2_, shows a strong cooperativity (dimeric binding) and is approximately 200 times stronger than that of f-PlnD. This result is consistent with previous transcription analysis, which showed that transcription of the *plnABCD *operon is one of the first to be induced, and that its expression is maintained at a high level throughout the whole period of induction [16]. From the same study it was shown that at the end of induction, transcription of *plnABCD *was turned off at a time-point later than that of the other operons. This indicates that PlnC binds to P_*plnA *_during the initial steps of bacteriocin production, without being seriously interfered by PlnD, in order to boost the expression of the regulatory operon. Consequently, repression of bacteriocin production by PlnD is less likely to occur through competition for the regulatory promoter P_*plnA *_(assuming that the *in vivo *levels of PlnD do not markedly exceed that of PlnC). In contrast, the negative regulator f-PlnD displayed higher affinity (K_D2_) for P_*plnM *_and P_*plnG*_, binding approximately 40 and 3 times stronger than f-PlnC, respectively. Interestingly, transcription from these two promoters have been shown to deviate from the other promoters, i.e., induction of *plnMNOP *(encoding proteins with unknown function in bacteriocin biosynthesis) has been shown to be delayed by 2 hours compared to *plnABCD*, while *plnGHSTUVW *(transport operon) is the first to be down-regulated (approximately 2 hours before *plnABCD*) [16]. One could speculate that PlnD's stronger binding to P_*plnG *_could result in repression of *plnGHSTUVW *expression, eventually leading to reduced IP maturation and secretion which in turn would shut down the whole system. However, since elevated transcription of the transport operon has been observed in previous studies [16], and because it encodes components (PlnGH) that are crucial for induction of the *pln *locus, repression of *plnGHSTUVW *by PlnD must somehow be circumvented at the initial phase of induction, e.g., by different activity and/or levels of the two RRs during bacteriocin production. It is worth to note that *plnD *contains the start-codon TTG, which is less frequently used in prokaryotes than the start-codon GTG in *plnC *[24]. Whether the different start-codons in *plnC *and *plnD *could result in different levels of PlnC compared to PlnD during bacteriocin production is yet to be determined.

Regarding the binding of f-PlnC and f-PlnD to P_*plnJ *_(*plnJKLR*; bacteriocin operons) it was somewhat difficult to determine which of the two RRs displayed highest affinity for this promoter. Although f-PlnC seems to associate to P_*plnJ *_at a slower association-rate than f-PlnD (Figure 2), f-PlnC also dissociated at a much slower rate. Consequently, we could not tell which of the regulators that bound P_*plnJ *_with highest affinity. On the other hand it is clear that this promoter displayed much higher binding capacity for f-PlnD than for f-PlnC (130 RU and 20 RU, respectively), indicating that there are factors limiting the amount of f-PlnC that can bind to this promoter, but that once bound, the strength of the interaction is not necessarily weaker than that of f-PlnD. As for binding to the second bacteriocin promoter P_*plnE *_(*plnEFI*), our binding data suggest that f-PlnC binds strongly to this promoter, while binding of f-PlnD could hardly be detected. However, previous gel-shift analysis have shown that PlnD also can bind to this promoter, though differently than PlnC [11]. The reason responsible for this discrepancy is unclear.

A recent study by Francke and co-workers (2008) employed sequence alignments and information from three-dimensional structures to show that conserved differences in the DNA-binding regions of transcriptional regulators can be correlated to specific variations in their cognate target sites [25]. A similar approach was used to look for specific differences in the DNA-binding domains of PlnC and PlnD that might explain their different binding properties, using amino acid sequence alignments and the solved structure of the *Staphylococcus aureus *AgrA LytTR domain [14] as a guide; like AgrA, PlnC and PlnD contain a LytTR-like DNA binding domain. However, the amino acids in PlnC and PlnD which correspond to the DNA-binding residues in AgrA were identical between PlnC and PlnD, suggesting that other differences located outside the DNA binding sequence might be responsible for their different binding properties.

Together with previous transcriptional analysis, the binding kinetics presented in this study indicates that other mechanism(s) in addition to the promoter affinity of PlnC and PlnD also are involved in controlling their opposite functions during bacteriocin production. However, the exact process behind this mechanism is beyond the scope of this study and requires further experimental studies. Nevertheless, by characterising the binding kinetic properties of these two RRs, we have gained important information regarding their interactions with the *pln *promoters, which will be of great value in the future in order to solve the puzzle of how these antagonising RRs regulate expression of the *pln *locus. To our knowledge this is the first report describing the DNA-binding kinetics of two RRs that belong to the important LytTR family, which includes numerous regulators involved in virulence development such as production of toxins in *Staphylococcus aureus *(AgrA) and *Clostridium perfringens *(VirR) and biofilm formation in *Pseudomonas aeruginosa *(AlgR) [26].

## Conclusion

In present study we have used SPR spectroscopy to obtain quantitative information regarding the DNA-binding kinetic properties of two highly homologous but counteracting RRs, PlnC and PlnD [11,15,16]. It was revealed that these co-expressed RRs indeed bind to the same promoters with different affinities as well as different degrees of cooperativity. Most importantly, the activator PlnC was shown to bind much stronger to the regulatory promoter P_*plnA *_compared to that of the repressor PlnD. This result suggests that PlnC binds P_*plnA *_without much competition from PlnD in order to activate transcription of *plnABCD*, which in turn activates the whole *pln *locus. PlnD, on the other hand, displayed stronger binding to P_*plnG *_(controlling the transport operon *plnGHSTUVW*), which might function as a mechanism of down-regulating the *pln *locus. By repressing the production of the transport system, it would result in depletion of the extracellular IP and consequently lower levels of *plnABCD*. However, the mechanism responsible for why repression of *plnGHSTUVW *by PlnD is avoided at the early stage of induction requires further experimental studies.

## Methods

### DNA and protein preparation

N-terminally flag-tagged PlnC and PlnD (f-PlnC and f-PlnD) were expressed in *L. sakei *Lb790 [27] using the SIP system [28], and purified by immunoprecipitation as described by Straume et al [29]. The proteins were eluted at >80% purity as judged by SDS-PAGE stained with Coomassie blue. The purified proteins were stored on ice in 20 mM Tris-HCl, pH 7.4 containing 0.1 mM phenylmethylsulphonyl fluoride, and used for binding studies within 24 hours.

HPLC-purified and biotinylated DNA 50-mers containing the two tandem repeats of the five *pln *promoters, P_*plnA*Δ*L *_with a deleted left repeat, and the control DNA were purchased from Invitrogen. For making P_*plnA*Δ*L*_, the left repeat of P_*plnA *_was changed from CACGTTTAA to T*TAAAATT*T so that it no longer contains any conserved residues on this region. An amount of 1 pmol biotin-labelled forward strand (Table 1) was annealed to 10 pmol of its complementary reverse strand by heating to 95°C and gradually cooling to room temperature. The annealing step was performed in 20 mM Tris-HCl, pH 7.4 containing 5 mM MgCl_2_. The 50-mer duplexes were stored at -20°C.

### Surface plasmon resonance spectroscopy

Interaction between the five *pln *promoter sequences and either of the RRs f-PlnC or f-PlnD was monitored using SPR spectroscopy. All binding experiments were carried out on a Biacore3000 SPR biosensor, using DNA-immobilised SA-chips. Briefly, sensor-chips coated with streptavidin were treated with 1 M NaCl in 40 mM NaOH to prepare the streptavidin surface for binding of biotin-labelled DNA. Biotinylated DNA-duplexes were diluted to 5 nM in HBS-EP buffer (150 mM NaCl, 3 mM EDTA, 0.005% P-20, 10 mM HEPES, pH 7.4), and injected to the sensor chip at a flow-rate of 20 μl/min. By calculating the sensor signal, 500 RU were immobilised onto the chip surface, followed by washing (20 μl/min) with HBS-EP buffer for 1 min. To correct for non-specific binding, control DNA (see Table 1) containing non-consensus regulatory repeats was immobilised in the first flow cell on each sensor chip. In another control, we also confirmed that both the flag-peptide used to elute the proteins and the purified RRs had no significant binding to the sensor-chip surface when the chip was not coated with DNA.

Prior to binding assays, the purified proteins were diluted in binding buffer containing 100 mM NaCl, 5 mM MgCl_2_, 0.025% Triton X-100, 1 mM EDTA, and 20 mM Tris-HCl, pH 8.0. This was done to retain the activity of f-PlnC and f-PlnD. The buffer system in the SPR instrument was changed to binding buffer for optimised binding conditions and to reduce the bulk refractive index between samples and running buffer. Following dilution of protein samples to appropriate concentrations, they were injected to the flow cells. The DNA-protein association was allowed to proceed for 120 seconds followed by dissociation for 180 seconds at a flow-rate of 50 μl/min. The temperature was kept constant at 25°C in all runs. After each run, the surface of the sensor chip was regenerated by injecting 1 M NaCl for 1 min, followed by washing with binding buffer for 1 min at 50 μl/min. All sensorgrams were analysed for bulk drift and mass transfer limitations.

### Data analysis

All binding data were corrected for non-specific binding between the RRs and the control DNA and for bulk refractive index. The binding kinetics was calculated globally using the BIAevaluation software version 4.1, except for binding of f-PlnC to P_*plnG *_which was estimated by local fitting of the association phase. The fitting model called heterogeneous ligand – parallel reactions provided the best fit for the ligands containing two binding sites on the DNA molecule. This model is based on interactions between two independent ligands (A) and one analyte (B), in this case the two repeats on DNA function as the two ligand sites and the RR as the analyte. The formula for this model is:



where *ka1 *and *ka2 *represent the association rate to the first and second ligand site, while *kd1 *and *kd2 *values represent the dissociation rate from the first and second site, respectively. *ka *relates the rate at which the ligand and analyte associate to the concentration of molecules (*ka *[A] [B]), while *kd *relates the rate at which the complex dissociates to the concentration of the complex (*kd *[AB]). The *ka *has the unit of M^-1^t^-1 ^where M is molarity and t is time, and *kd *has the unit of t^-1^. The dissociation constant K_D _is calculated by dividing *kd *by *ka*, giving K_D _the unit of M.

For the ligand containing only one binding site (P_*plnA*Δ*L*_), the Langmuir 1:1 fitting model provided the best fit. The formula for this model is:



The accuracy of the fits was evaluated by using residual plots and reduced chi-square (χ^2^) values.

## Abbreviations

bp: base pairs; C11-*pln*, WCFS1-*pln*, NC8-*pln*, J23-*pln*, J51-*pln*: bacteriocin locus in the five *L. plantarum *strains C11, WCFS1, NC8, J23 and J51; IP: inducer peptide; HPK: histidine protein kinase; RR: response regulator; RU: response units; SPR: surface plasmon resonance.

## Authors' contributions

DS contributed with experimental design, performance of all experiments, and writing of the manuscript. RFJ and MB participated in initial experimental design and SPR spectroscopy and assisted with the data analysis. DBD and IFN contributed with supervising and writing of the manuscript. All authors read and approved the final manuscript.

## Supplementary Material

Additional file 1 **Genetic organisation of the *pln *loci from *L. plantarum *C11, WCFS1, J51, NC8 and J23**. The figure provided shows the genetic organisation of the *pln *locus found in *L. plantarum *C11, WCFS1, J51, NC8 and J23. Click here for file
