# Supplementary material for: DNA binding kinetics of two response regulators, PlnC and PlnD, from the bacteriocin regulon of Lactobacillus plantarum C11
Source: BMC Biochem. 2009 Jun 11;10:17. doi: 10.1186/1471-2091-10-17 (PMC2714321; doi:10.1186/1471-2091-10-17)
Supplement: Additional file 1 — Genetic organisation of the pln loci from L. plantarum C11, WCFS1, J51, NC8 and J23. The figure provided shows the genetic organisation of the pln locus found in L. plantarum C11, WCFS1, J51, NC8 and J23. [file 1471-2091-10-17-S1.doc]

Supplementary material:

**
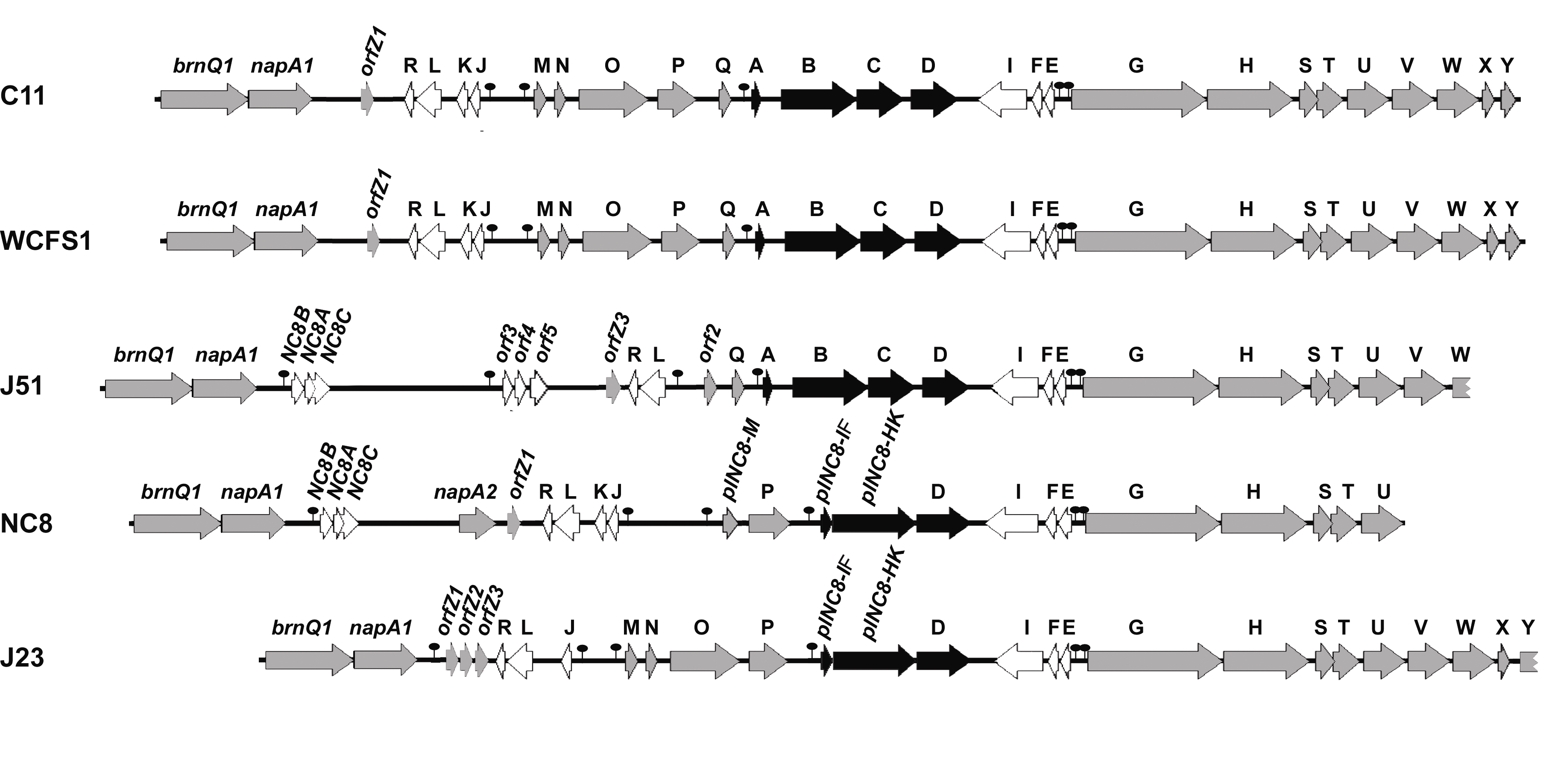
**

**Figure S1.** **Genetic organisation of the *pln* loci from *L. plantarum* C11, WCFS1, J51, NC8 and J23.** The regulatory operons are shown in black and the bacteriocin encoding operons in white. The tandem repeats located in the promoters of the regulated operons are indicated by hairpins.
